# Sleep‐related disorders in children: A narrative review

**DOI:** 10.1002/pdi3.76

**Published:** 2024-04-27

**Authors:** Yogev Cohen, Joel Reiter, Alex Gileles‐Hillel

**Affiliations:** ^1^ Faculty of Medicine The Hebrew University of Jerusalem Jerusalem Israel; ^2^ Pediatric Pulmonology and Sleep Unit, Pediatric Department Hadassah Medical Center Jerusalem Israel; ^3^ The Wohl Institute for Translational Medicine Hadassah Medical Center Jerusalem Israel

**Keywords:** insomnia, pediatrics, sleep, sleep apnea

## Abstract

Sleep‐related disorders in children can significantly impact children's physical, emotional, and cognitive development and constitute a major source of parental concern. This comprehensive review aims to describe sleep‐related disorders commonly encountered in pediatric practice, their etiology, diagnosis, and management strategies. The review explores various disorders prevalent in different age groups, including insomnia, sleep apnea, parasomnias, and circadian rhythm disorders, highlighting the importance of early identification and intervention for optimal child health and well‐being.

## 1 INTRODUCTION

Sleep is crucial for the developing brain during early childhood. The intricate organization and regulation of sleep and wakefulness involve complex interactions among various components of the central nervous system (CNS). Sleep is a fundamental physiological process that plays a crucial role in the growth, development, and overall well‐being of children.^1^, ^2^ By the time a child reaches the age of 2 years, they have spent roughly 9500 h (equivalent to 13 months) sleeping, which exceeds the 8000 h they have spent on all other activities while awake.^3^, ^4^ Between the ages of 2 and 5 years, the balance between a child's waking and sleeping hours gradually evens out.^3^ Throughout childhood and adolescence, sleep continues to account for approximately 40% of a child's average day. Many children experience sleep‐related disorders that can disrupt their sleep patterns and affect various aspects of their lives. Sleep disorders in children are multifaceted and arise from various underlying factors that have physiological, behavioral, psychological, and environmental components. Understanding and addressing these disorders is essential for promoting healthy sleep habits and optimal child health.^3^, ^4^, ^5^, ^6^, ^7^

Sleep architecture and behaviors undergo significant changes as children develop.^8^, ^9^ For example, infants typically achieve sleep consolidation (continuous nighttime sleep augmented by short daytime naps) and regulation (the ability to fall asleep without intervention) milestones around 6 months of age, allowing them to sleep longer at night and self‐soothe.^8^, ^9^ Parental awareness and reporting of sleep problems may vary across childhood, and the definition of a “sleep problem” can be subjective. Lastly, sleep patterns can differ among cultures.^3^, ^4^

The range of normal sleep duration by age can be seen in Table 1.^3^

**TABLE 1:** Normal sleep duration ranges by age.

Age group	Mean sleep duration (hours)	Range (hours)	Normal sleep physiology and development
0–2 months	14.6	9.3–20.0	Three sleep states: Active (“REM‐like,” 50% of sleep), quiet (“non‐REM‐like”), and indeterminate. Enter sleep through an active state. Three amounts of active/REM sleep declines.
Around 3 months	13.6	9.4–17.8	
Around 6 months	12.9	8.8–17.0	
Around 12 months	12.9	10.1–15.8	
1–2 years	12.6	10.0–15.2	REM sleep amounts continue to decline. Sleep cycles every 90 min. High levels of SWS.
2–3 years	12	9.7–14.2	
4–5 years	11.5	9.1–11.4	
Around 6 years	9.7	8.1–11.4	
Around 7 years	9.4	7.9–10.9	Latency from sleep onset to REM sleep increases. High sleep efficiency (time asleep/time in bed). 40% decline in SWS. REM sleep at adult levels (25%–30%).
Around 8 years	9.3	7.8–10.8	
Around 9 years	9.3	7.8–10.8	
Around 10 years	9.1	7.6–10.7	
Around 11 years	9	7.3–10.6	
Around 12 years	8.8	7.3–10.4	

Abbreviations: REM, rapid eye movements; SWS, slow wave sleep.

## 2 SLEEP DISORDERS IN NEWBORNS, INFANTS, AND TODDLERS

Newborns have irregular sleep patterns and often experience day and night reversal that may persist for several months. Perceived sleep disorders may therefore result from inappropriate parental expectations.^10^ However, by 2–3 months, a more regular rhythm of sleepiness and alertness emerges.^1^, ^3^, ^5^, ^7^ This may be encouraged by differentiating day and night sleep by differences in light exposure activity, environment, etc.^11^

A safe sleeping environment may be considered the most important issue in this age group. The most recent update of the American Academy of Pediatrics (AAP) recommended supine positioning, a firm, non‐inclined sleep surface, room sharing without bed sharing, avoidance of soft bedding and overheating, as well as additional recommendations given in their 2022 statement.^12^

Temporary disturbances to normal sleep often occur in association with developmental milestones or acute illnesses, and they tend to resolve on their own. Some parental reinforcing behaviors may prolong the acute sleep disturbance, such as excessive rocking.^3^, ^13^ However, not all sleep problems in infants are short‐lived. Predisposing factors, such as an inability to self‐soothe, maternal depression, and bed‐sharing, increase the risk of chronic sleep disorders.^3^ Approximately 25%–50% of 6‐ to 12‐month‐olds, and 30% of 1‐year‐olds, experience problematic night wakings, while around 50% have difficulties with sleep onset or settling at 12 months.^3^, ^4^

Behavioral extinction techniques can be employed to address sleep disorders in infants. These techniques involve gradually reducing or eliminating certain sleep‐related behaviors, such as night‐time feedings or excessive comforting, in order to encourage the development of independent and self‐soothing sleep habits.^14^ By gradually removing the reinforcement for undesired behaviors, infants learn to self‐soothe and establish healthy sleep patterns over time. There are several methods for addressing behavioral sleep disorders in infants: unmodified extinction, extinction with parental presence, and graduated extinction. All of these involve not responding to the child's calls or cries once they have been put to bed. Some methods include brief entries into the child's room to settle them down with minimal interaction, avoiding actions such as picking up the child, cuddling, engaging in conversations, or offering food.^14^

### 2.1 Night waking

Night wakings are a common sleep issue experienced by infants and toddlers.^6^, ^7^, ^15^ Most healthy infants are capable of sleeping through the night by 3 months of age and no longer require nighttime feedings by 6 months. Still, a significant percentage (25%–50%) continue to wake up during the night at 9–12 months.^16^, ^17^ It should be noted that healthy children can wake up spontaneously several times during the night and can fall back to sleep, often without being aware of the awake period.^18^ Problems arise when a child is unable to go back to sleep, often due to inappropriate sleep‐onset associations or insufficient parental limit setting. Associations are conditions or behaviors the child associates with falling asleep at bedtime and requires during the night to return to sleep after natural awakenings. Parental responses to night wakings, such as soothing, bed sharing, rocking, or feeding, can reinforce these behaviors and contribute to their persistence.^13^, ^18^ Establishing consistent self‐soothing skills, limit‐setting to unwanted behaviors, and minimizing parental reinforcement of night wakings are key to reducing these behavioral sleep disruptions in infants and toddlers.

### 2.2 Sleep‐related rhythmic movements

Head banging, body rocking, and head rolling during the transition to sleep are all behaviors categorized as sleep‐related rhythmic movement conditions. These involve repetitive, rhythmic movements of large muscle groups and typically occur during the transition to sleep, nap times, and following nighttime arousals, serving as self‐soothing mechanisms.^3^ While these behaviors are usually benign, unless they cause clinical consequences or impair daytime functioning, they may be distressing to caregivers. Children with psychiatric or neurological disorders may be at a higher risk of injury due to the intensity and frequency of these behaviors.^19^ No treatment is usually required beyond reassurance and ensuring the safety of the sleeping environment.^3^, ^4^

## 3 SLEEP DISORDERS IN CHILDREN AGED 3–12

Difficulties falling asleep and night‐waking are prevalent in this age group, affecting 15%–30% of children.^3^, ^4^ Acute sleep problems can become chronic ‐ in one study, 84% of children aged 15–48 months with bedtime struggles and/or night‐waking continued to experience significant sleep disturbances by the age of 3 years.^3^ It should be noted that some sleep disorders in this age group may arise earlier (e.g., night terrors), and others may affect also older children and adolescents (e.g., obstructive sleep apnea). We discuss toddlers, preschoolers, and school‐aged children as a single group, although it should be noted that some sleep disorders are more common in specific age‐groups.

### 3.1 Nighttime fears

Nighttime fears in children are common and constitute a normal part of their development.

Studies show that nighttime fears are reported by 59% of 4‐ to 6‐year‐olds, 85% of 7‐ to 9‐year‐olds, and 80% of 10‐ to 12‐year‐olds.^20^, ^21^ These fears often emerge during preschool years when children become aware of potential dangers. The specific fears children experience vary based on their age, ranging from imaginary creatures to more realistic concerns like burglars or natural disasters. Environmental threats and personal security are the most common sources of fear. In severe cases, children may refuse to go to bed, sleep without lights, or be alone in dark places, leading to a need for constant companionship or shared sleeping arrangements.^5^, ^15^, ^20^, ^21^

### 3.2 Nightmares

Nightmares are intense and distressing dreams that can awaken children and adolescents, leaving them in a state of fear and requiring comfort. These vivid dreams often involve threats to survival, security, and physical well‐being.^20^, ^21^ Nightmares occur during rapid eye movement (REM) sleep and typically involve feelings of fear, anxiety, and other negative emotions. The content of nightmares varies with age, reflecting developmental concerns and experiences.^3^ Young children may fear separation from their parents or encountering imaginary creatures, while older children may have nightmares related to movies, stories, or real‐life distressing events. Nightmares can be triggered by stressors or traumatic experiences and may leave children hesitant to return to sleep.^3^

### 3.3 Disorders of arousal

Parasomnias are sleep disorders characterized by abnormal behaviors or experiences that occur during sleep or the transition from wakefulness to sleep.^22^ They are classified into two main categories: non‐rapid eye movement (NREM) related parasomnias and REM related parasomnias such as nightmares described above. NREM‐related parasomnias are also termed disorders of arousal.^22^

NREM‐related parasomnias include confusional arousals, sleepwalking, and sleep terrors. They typically occur during deep sleep stages, particularly slow‐wave sleep, which is characterized by high brainwave activity associated with deep restorative sleep.^3^, ^22^, ^23^

Confusional arousals are episodes of disorientation and unresponsiveness to the environment upon awakening. They often occur when a child is abruptly woken up, especially early during the night or when trying to wake up in the morning. During confusional arousals, children may sit up in bed, exhibit agitated or combative behaviors, and may have difficulty recognizing or interacting with their surroundings. Confusional arousals are estimated to affect approximately 17% of children aged 3%–13% and 2.9%–4.2% of adults older than 15.^24^ They often co‐occur with sleepwalking and sleep terrors, typically starting before age 5 and lasting up to 13 years of age but are distinguished in that the child remains in bed.^24^

Sleepwalking involves complex behaviors performed while the individual is still asleep. Children experiencing sleepwalking may appear confused, dazed, or inattentive. Their eyes are typically open, and they may mumble or give nonsensical answers if spoken to. Sleepwalkers can engage in various activities ranging from simple movements to more complex actions like going downstairs, leaving the house, or engaging in potentially risky behaviors. Injuries can occur during sleepwalking episodes, making safety a concern for caregivers. Sleepwalking is observed in many children, with prevalence rates of around 15%–40% for at least one episode.^22^, ^25^ About 17% of children regularly sleepwalk, while 3%–4% experience frequent episodes. Sleepwalking can persist into adulthood, affecting approximately 4% of adults. The prevalence is higher in children with a family history of sleepwalking.^25^

Sleep terrors, also known as night terrors, are intense episodes of fear and autonomic arousal that occur during sleep. They are often accompanied by dramatic manifestations such as screaming, rapid breathing, increased heart rate, and physical agitation. Despite the alarming nature of sleep terrors, the child remains unaware and unresponsive to their surroundings. Sleep terrors can be distressing for parents or caregivers who witness them but are generally less traumatic for the child than a nightmare or bad dream. Sleep terrors are reported in approximately 1%–6.5% of children, usually during the preschool and elementary school years. Most individuals outgrow sleep terrors by adolescence.^26^

These disorders of arousal typically occur within a few hours after sleep onset, lasting for several minutes to around 30–40 min.^20^, ^21^ Amnesia for the episode is common, with the child often having no recollection of the events upon awakening.

Sleep deprivation, Restless Legs Syndrome (RLS), and obstructive sleep apnea (OSA) are the most common modifiable triggers for parasomnias in children. Treatment of these disorders may reduce or resolve parasomnias in affected children.^27^

### 3.4 Bruxism

Bruxism is the involuntary grinding or clenching of teeth during sleep. It typically occurs during non‐REM sleep stages, mainly stages 1 and 2, and less frequently during REM sleep. While occasional teeth grinding is common, persistent bruxism is less frequent.^28^

In young children, bruxism often doesn't require treatment. However, if older children or adolescents experience dental damage or persistent jaw pain, it may be necessary to seek a dentist's referral for a nighttime intraoral appliance. Prevalence rates range from 3.5% to 49.6% in children and adolescents, with most studies reporting rates of 14%–17% in children and 12% in adolescents. Bruxism often persists into adulthood in about two‐thirds of cases and keeps on decreasing as the patient gets older.^28^, ^29^

### 3.5 Nocturnal enuresis

Nocturnal enuresis, or bedwetting, is characterized by involuntary voiding during sleep. It is diagnosed when it occurs at least twice a week after the age of 5 years, and both primary (never had a dry period of 6 months) and secondary (previously dry for 6 months but now bedwetting at least twice a week) forms exist. Enuresis can occur in any sleep stage, with most episodes happening in the first half of the night. In childhood, boys are three times more likely to experience enuresis compared to girls, primary enuresis gradually decreases with age to around 1%–2%.^30^

The prevalence of nocturnal enuresis is increased in children with OSA.^24^, ^30^ It is therefore important to investigate for underlying OSA in children in whom enuresis worsens with age, rather than improving.^30^

### 3.6 Sleep‐related breathing disorders and obstructive sleep apnea

A complete review of pediatric OSA is beyond the scope of this review. OSA is part of a range of sleep‐related breathing disorders, and it is characterized by pauses in breathing during sleep, with a significant reduction or complete cessation of airflow.^31^, ^32^ We describe OSA here, as it is most prevalent in this age group, but it is important to remember that children can suffer from OSA at any age. Therefore, pediatricians should be familiar with the presentation of OSA and screen for it.^33^ The prevalence of pediatric OSA is estimated to be between 1% and 5%, with slightly higher rates when defined by parent‐reported symptoms. Sleep studies that monitor ventilation indicate an overall prevalence of 1%–4%, with a narrower range of 2%–3% using stricter criteria. Certain pediatric populations are affected by OSA more than the general population. These include children with obesity, trisomy 21, a history of prematurity, sickle cell anemia, and craniofacial syndromes among others.^31^, ^34^ Children at risk for OSA generally have a narrowed upper airway due to factors such as adenotonsillar hypertrophy (the most common cause), intraluminal fat deposits, reduced pharyngeal muscle tone, and decreased central ventilatory drive. Additionally, upper airway stability, arousal threshold, and the collapsibility of the airway during sleep play important roles in the pathophysiology of OSA.^31^, ^34^, ^35^ Several screening tools exist for primary care use, but any combination of snoring, restless sleep, persistent rhinorrhea, and behavioral difficulties should raise the suspicion of OSA.^36^ The gold‐standard diagnostic modality is full polysomnography (PSG), although a diagnosis of OSA can be made without it, based on high clinical suspicion.^37^ Adenoidectomy with or without tonsillectomy is the mainstay of treatment for pediatric OSA. Additional treatment options include anti‐leukotrienes and intranasal steroids for well‐defined mild cases and continuous positive airway pressure for recalcitrant cases.^38^

### 3.7 Restless legs syndrome

RLS is a neurological disorder characterized by an irresistible urge to move the legs accompanied by uncomfortable sensations. These symptoms are usually relieved by movement and tend to worsen during periods of rest or inactivity. Restless Legs Syndrome often has a circadian component with symptoms peaking in the evening.^39^, ^40^ The prevalence of pediatric RLS is 1%–6% with possible higher rates in children on the autistic spectrum. Low serum ferritin levels have been associated with RLS in children, and iron treatment may be effective in eliminating RLS.^41^

## 4 SLEEP DISORDERS IN ADOLESCENCE

Several sleep‐related disorders arise in adolescence. One of the most common is chronic sleep deprivation.^3^, ^4^ This condition can have significant neurobehavioral consequences, negatively affecting mood, vigilance, reaction time, attention, memory, behavioral control, and motivation. Additionally, adolescents are prone to various sleep disorders, with at least 20% experiencing significant sleep problems.^42^

### 4.1 Circadian rhythm disorders

Adolescents may experience circadian rhythm disorders when there is a mismatch between their internal sleep‐wake rhythm and the demands imposed by their daily schedules, resulting in challenges in establishing a consistent and beneficial sleep pattern.^43^, ^44^ The most common circadian sleep‐wake disorder in the pediatric population is delayed sleep‐wake phase disorder (DSWPD), characterized by a persistent shift of over 2 h in the sleep‐wake schedule. Individuals with DSWPD have trouble falling asleep at a desired bedtime and struggle to wake up in the morning, resulting in daytime sleepiness.^43^, ^44^ They may rely on lengthy afternoon naps or try to compensate on weekends. Shifting these children's sleep schedule to an earlier set point without external assistance is challenging even for motivated adolescents. However, setting bedtimes for adolescents has been shown to have positive effects.^42^, ^45^ Furthermore, early school times are a major contributor to adolescents' sleep deprivation and the ensuing psychological and physical morbidities. Later school start times have been shown to reduce sleepiness, improve academic performance, reduce involvement in car accidents, and promote healthier eating behaviors, among others.^46^, ^47^ The American Academy for Sleep Medicine and other health organizations in the US recommend that middle school and high school start times should be 8:30 AM or later.^46^

### 4.2 Insomnia

Insomnia refers to significant and persistent difficulties with sleep initiation, maintenance, or early morning awakening.^48^, ^49^, ^50^ These sleep complaints are accompanied by impaired daytime functioning, which can manifest as irritability, difficulty concentrating, behavioral disturbances, mood changes, and academic performance issues. Insomnia can be categorized as short‐term or transient, typically related to acute stressful events, or long‐term and chronic, persisting for at least three nights per week over a period of 3 months or more.

Insomnia is a complex disorder, with genetic factors as well as biological, environmental, and social factors all contributing to its development. Some of the significant factors contributing to the development and persistence of insomnia in adolescence include female sex and puberty, school stress, electronic media use, and high caffeine intake.^48^, ^49^, ^51^

It is important to differentiate insomnia from other sleep disorders that may contribute to sleep difficulties, such as RLS or OSA.^32^, ^48^ Sometimes, the symptoms of these underlying sleep disorders or those resulting from circadian misalignment and sleep deprivation can overlap with those of insomnia, making it necessary to conduct a thorough evaluation to identify the primary cause.^3^, ^40^, ^48^

Insomnia in adolescents can present differently than in adults. Parents or caregivers may report concerns about “bedtime problems” or “night‐wakings” rather than specifically labeling it as insomnia. The manifestations of insomnia in adolescents may include resistance at bedtime, hyperactivity, academic difficulties, and other behavioral problems that stem from sleep deprivation and Excessive daytime sleepiness (EDS).^48^, ^52^

The diagnosis of insomnia is made based on the presence of sleep complaints, impairment in functioning, and distress related to poor sleep, without necessarily attributing it to a specific comorbid condition.^50^, ^53^

Sleep diaries and actigraphy are useful to characterize sleep timing and duration, assisting in the diagnosis of insomnia and circadian rhythm disorders.

There are two main approaches for the treatment of primary insomnia disorder in adolescence: cognitive behavioral interventions, and pharmacotherapy, although alternative methods such as acupuncture have also been reported to improve insomnia symptoms. These can be used in isolation or as a combination. To date, research into the effectiveness of treatments in the adolescent population is largely lacking.^51^

### 4.3 Hypersomnia and narcolepsy

Excessive daytime sleepiness refers to the inability to stay awake and alert during the major waking periods of the day, leading to uncontrollable urges to sleep or unintended lapses into drowsiness or sleep. It is important to distinguish EDS from fatigue, as EDS is characterized by a propensity for sleepiness, although the distinction may not always be clear based on history alone.^54^

Excessive daytime sleepiness can stem from primary sleep disorders with inadequate or disrupted sleep, such as OSA, insomnia, RLS, and periodic limb movement disorder, or from behavioral factors that curtail sleep.^3^, ^54^, ^55^

There are also less common but significant causes of EDS associated with hypersomnia of CNS origin. One notable example is narcolepsy, a lifelong CNS disorder that typically emerges during adolescence or early adulthood. Narcolepsy is characterized by profound daytime sleepiness and significant functional impairment. Additional symptoms often include cataplexy (sudden loss of muscle control), hypnagogic or hypnopompic hallucinations (vivid dream‐like experiences when falling asleep or waking up), sleep paralysis, automatic behaviors, and disturbed nocturnal sleep.^3^, ^55^ Narcolepsy can be further classified into narcolepsy with cataplexy (type 1: characterized by hypocretin deficiency), narcolepsy without cataplexy (type 2), and secondary narcolepsy due to medical conditions.

Central hypersomnias encompass both chronic and persistent disorders like narcolepsy and idiopathic hypersomnia, as well as episodic and recurrent disorders like Kleine‐Levin syndrome.^56^, ^57^

In adolescents with narcolepsy, the most prominent manifestation is profound EDS, characterized by an increased baseline level of daytime drowsiness and repeated sudden sleep episodes that can occur even during engaging activities. These sleep attacks are described as irresistible, with individuals unable to stay awake despite their best efforts. The episodes are relatively short (10–20 min) and are often reported as temporarily refreshing in adults. Individuals with narcolepsy may also experience brief, unaware sleep episodes known as “microsleeps,” which can lead to automatic behaviors and memory lapses.^54^, ^56^, ^57^

Narcolepsy typically begins in adolescence or early adulthood, although symptoms may initially manifest in school‐aged children or even younger. However, the early signs of narcolepsy are often overlooked, misinterpreted, or misdiagnosed as other medical, neurological, or psychiatric conditions, leading to delayed diagnosis.^55^, ^56^, ^58^

The prevalence of EDS in school‐aged children and adolescents is between 10% and 20%, although the exact figures vary depending on the definition of EDS used. On the other hand, the prevalence of narcolepsy in this age group is reported to be between 3 and 16 per 10,000 individuals, with some variability between ethnic and racial groups.^59^ The incidence rates for narcolepsy with cataplexy and narcolepsy without cataplexy are estimated at 0.74 and 1.37 cases per 100,000 person‐years, respectively. Narcolepsy without cataplexy accounts for about half or less of all narcolepsy cases (10%–50%).^3^, ^54^, ^55^

## 5 SLEEP DISORDERS IN SPECIFIC PEDIATRIC POPULATIONS

Sleep disorders are highly prevalent in children with neurodevelopmental disabilities, such as autism and various congenital or inherited conditions.^3^, ^4^ Although the types of sleep disorders seen in these children are not unique, their frequency and severity are often higher compared to typically developing children.^3^ Sleep disorders in this population tend to be chronic, resistant to treatment, and more likely to recur.^3^, ^24^ It is common for multiple sleep disorders to coexist in these children, and the impact of disrupted or insufficient sleep on their cognitive, emotional, and social development, and their behavior, can be substantial, considering their already vulnerable nature.

Below we describe some selected, more common conditions of specific pediatric populations with an increased prevalence of sleep disorders.^60^

### 5.1 Obesity

Obesity is a modern‐day epidemic affecting about 17% of the pediatric population in the US at various ages from infancy to adolescence.^61^ There is a reciprocal association between the two conditions: poor sleep quality and short sleep duration predisposes to weight gain and obesity. Conversely, obese children and adolescents experience poor sleep more frequently than their non‐obese counterparts and have a higher prevalence of sleep‐disordered breathing problems.^62^

The most common sleep‐related disorder in children with obesity is OSA, with a prevalence of 20%–40%.^63^ Obese children have a unique OSA phenotype, accompanied by increased susceptibility to cardio‐metabolic consequences.^64^, ^65^ Due to the increased prevalence and morbidity of OSA in obese children and adolescents, it is important to maintain a high index of suspicion for screening. The first line of treatment for obese‐related pediatric OSA is adenotonsillectomy, similar to non‐obese children. Obesity is a significant risk factor for residual OSA post‐surgery, often requiring the use of additional treatment modalities like continuous airway pressure support.^66^

### 5.2 Trisomy 21

Studies of sleeping patterns of children with Trisomy 21 have shown symptoms that are most consistent with insomnia. These children experience poor sleep quality, difficulties with falling asleep, bedtime resistance (refusal to go to bed or stay in bed), and nocturnal awakenings, as well as EDS. While these complaints are common in typically developing children, they have been documented in children with Trisomy 21 at relatively later ages and with higher frequency.^67^

In school‐aged children with Trisomy 21, the most common complaint is disturbed sleep. In one study, 60% of the participants reported disrupted sleep with nighttime awakenings. A significant proportion of the children in that study required assistance in waking up in the morning, had difficulty getting out of bed, and experienced prolonged sleep inertia. It was further shown that these complaints were not related to sleep‐disordered breathing.^68^ Sleep disorders have an adverse impact on the development of children with Trisomy 21, such as delayed language development.^69^ The first line of treatment for behavioral sleep disorders in children with Trisomy 21 involves behavioral interventions, as described above for typically developing children.^70^

Sleep‐disordered breathing is also highly prevalent in this population, with OSA affecting half of children and most adults with Trisomy 21,^71^ emphasizing the importance of screening. Unlike typically developing children, snoring and observable apneas may not be as prominent in children with Trisomy 21. Accordingly, the American AAP guidelines recommend a scheduled PSG before the age of 4 years for children with Trisomy 21, even in the absence of symptoms.^72^

Finally, children with Trisomy 21 have an increased prevalence of parasomnias, such as sleep talking and bedwetting.^67^, ^68^

### 5.3 Prematurity

Children born prematurely face an increased risk of neurodevelopmental issues, including language, motor skills, behavior, and self‐regulation difficulties. Some studies have found that preterm children may experience sleep disturbances, such as shorter daytime naps, reduced nighttime sleep duration, and poorer sleep quality during their first years of life.^73^ Sleep in preterm children may be less restful compared to their full‐term counterparts. Additionally, very low birth weight infants are at risk of reduced sleep efficiency at 8 years old and may have earlier sleep onset times in adulthood.^74^, ^75^ Sleep‐disordered breathing is also more prevalent in preterm children during middle childhood. These sleep disturbances have been linked to subsequent developmental issues, emphasizing the importance of adequate sleep for optimal infant development.^73^, ^75^ Mechanistically, sleep deprivation during critical periods of brain development can lead to cellular damage, oxidative stress in the hippocampus, suppression of neurogenesis, and affect brain areas responsible for motivation, attention, and goal‐directed behavior.

### 5.4 Autism spectrum disorder (ASD)

Children with neurodevelopmental disorders commonly experience subjective sleep problems, including insomnia and short sleep duration. Unlike typically developing children, these sleep disorders tend to persist and do not improve significantly with age.^76^ Parasomnias such as sleepwalking and movement disorders such as bruxism may be more prevalent, and there may be an increase in REM behavior disorder, a rare condition in childhood in which muscle atonia during REM sleep is absent, at times resulting in the acting out of dream content.^76^, ^77^ Behavioral interventions are the first‐line treatment approach for sleep disturbances in children with Autism spectrum disorder (ASD). These could be combined with melatonin pharmacotherapy. Potential adverse effects of melatonin and the lack of long‐term safety data should be discussed with caregivers.^78^, ^79^

## 6 TAKE HOME MESSAGE

Sleep and sleep disorders significantly impact the health and well‐being of children. Adequate and high‐quality sleep is essential for their physical, cognitive, and emotional development. Various sleep disorders can disrupt sleep patterns and have wide‐ranging consequences.

Recognizing the signs and symptoms of sleep disorders in children is crucial for early identification and appropriate management. A multidisciplinary approach ensures comprehensive care, accurate diagnosis, and tailored treatment plans for children with sleep disorders.

Promoting healthy sleep habits is key to preventing and managing sleep disorders in pediatric patients. Establishing consistent sleep routines, creating a conducive sleep environment, and promoting good sleep hygiene practices are essential for optimizing sleep quality and duration in children.

By prioritizing sleep health, identifying, and addressing sleep disorders promptly, and implementing comprehensive care strategies, pediatricians can enhance the overall well‐being and development of children.

## AUTHOR CONTRIBUTIONS

**Yogev Cohen**: Data collection, drafting of initial manuscript, review and editing. **Joel Reiter**: Data integration and draft review and editing. **Alex Gileles‐Hillel**: Data collection, drafting of initial manuscript, review and editing, serves as the guarantor of this work.

## CONFLICT OF INTEREST STATEMENT

Alex Gileles‐Hillel is the member of the Pediatric Discovery Editorial Board. To minimized bias, he was excluded from all editorial decision‐making related to the acceptance of this article for publication. The other authors have no confilict of interests.

## ETHICS STATEMENT

The study was a review of literature and therefore no ethical approval was needed.

## Data Availability

Data sharing is not applicable to this article as no new data were created or analyzed in this study.
